# Nitrate-Nitrogen Adsorption Characteristics and Mechanisms of Various Garden Waste Biochars

**DOI:** 10.3390/ma16165726

**Published:** 2023-08-21

**Authors:** Jingjing Yao, Zhiyi Wang, Mengfan Liu, Bing Bai, Chengliang Zhang

**Affiliations:** 1 Key Laboratory of Mine Ecological Effects and Systematic Restoration, Ministry of Natural Resources, Beijing 100081, China; yaojing1989_lucky@163.com; 2 Institute of Resources and Environment, Beijing Academy of Science and Technology, Beijing 100095, China; liumf9955@163.com (M.L.); baibing_1029@163.com (B.B.); zhang64@126.com (C.Z.); 3 The National Engineering Laboratory of Circular Economy (Industrial Wastewater Utilization and Industrial Water Conservation), Beijing 100095, China; 4 China Institute of Geo-Environment Monitoring, Beijing 100081, China

**Keywords:** nitrate-nitrogen, garden waste, biochar, pyrolysis temperature, NO_3_^−^–N removal efficiency

## Abstract

Nitrate-nitrogen (NO_3_^−^–N) removal and garden waste disposal are critical concerns in urban environmental protection. In this study, biochars were produced by pyrolyzing various garden waste materials, including grass clippings (GC), *Rosa chinensis Jacq*. branches (RC), *Prunus persica* branches (PP), *Armeniaca vulgaris Lam.* branches (AV), *Morus alba Linn.* sp. branches (MA), *Platycladus orientalis* (L.) Franco branches (PO), *Pinus tabuliformis Carrière* branches (PT), and *Sophorajaponica Linn*. branches (SL) at three different temperatures (300 °C, 500 °C, and 700 °C). These biochars, labeled as GC300, GC500, GC700, and so on., were then used to adsorb NO_3_^−^–N under various conditions, such as initial pH value, contact time, initial NO_3_^−^–N concentration, and biochar dosage. Kinetic data were analyzed by pseudo-first-order and pseudo-second-order kinetic models. The equilibrium adsorption data were evaluated by Langmuir, Freundlich, Temkin and Dubinin–Radushkevich models. The results revealed that the biochar yields varied between 14.43% (PT700) and 47.09% (AV300) and were significantly influenced by the type of garden waste and decreased with increasing pyrolysis temperature, while the pH and ash content showed an opposite trend (*p* < 0.05). The efficiency of NO_3_^−^–N removal was significantly influenced by the type of feedstock, preparation process, and adsorption conditions. Higher pH values had a negative influence on NO_3_^−^–N adsorption, while longer contact time, higher initial concentration of NO_3_^−^–N, and increased biochar dosage positively affected NO_3_^−^–N adsorption. Most of the kinetic data were better fitted to the pseudo-second-order kinetic model (0.998 > *R*^2^ > 0.927). Positive b values obtained from the Temkin model indicated an exothermic process of NO_3_^−^–N adsorption. The Langmuir model provided better fits for more equilibrium adsorption data than the Freundlich model, with the maximum NO_3_^−^–N removal efficiency (62.11%) and adsorption capacity (1.339 mg·g^−1^) in PO700 under the conditions of pH = 2, biochar dosage = 50 mg·L^−1^, and a reaction time of 24 h. The outcomes of this study contribute valuable insights into garden waste disposal and NO_3_^−^–N removal from wastewater, providing a theoretical basis for sustainable environmental management practices.

## 1. Introduction

Excessive nitrogen discharge into natural water bodies due to rapid industrial and agricultural development has led to widespread eutrophication, posing severe threats to both human and ecological health [1,2,3]. The leaching of nitrate-nitrogen (NO_3_^−^–N) is considered the most essential nitrogen loss pathway [4,5]. Nitrate fertilizers have been extensively used in agriculture since nitrogen is primarily absorbed in the form of nitrate ions, which can migrate to the surface and groundwater, thereby contaminating water resources [6]. This situation could be worse owing to the inappropriate treatment of wastewater [5]. For example, the concentration of nitrate in the groundwater of Yantai, China, was 17.80 mg·L^−1^ [7], surpassing the World Health Organization’s (WHO) drinking water limit of 10 mg·L^−1^ [8]. Therefore, the removal of NO_3_^−^–N has become a critical concern in water treatment strategies.

NO_3_^−^–N removal is a challenging task as nitrate ions exhibit strong stability and high solubility in water [8,9]. Currently, various methods are employed for removing nitrate ions from water, including physiochemical methods [10], biological technologies [3,11], membrane separation [12], and adsorption methods [5]. Among these, the adsorption method stands out as one of the most widely used methods because of its remarkable efficiency, cost-effectiveness, and high capacity [5,13]. In this context, biochar, produced through biomass pyrolysis, has received considerable attention as an effective adsorbent for treating pollutants, including nitrates. Its appeal lies in its straightforward production and operation, renewable nature, sustainability, affordability, eco-friendliness, high removal efficiency, and the abundance of surface functional groups that facilitate easy functionalization [1,8,14,15]. Moreover, biochar can be derived from a variety of materials, such as agricultural waste, garden waste, and animal waste sources [8,16,17,18]. Garden waste, a biodegradable byproduct resulting from the natural withering and artificial pruning of garden plants, such as grass clippings, leaves, branches, wood debris, and residual flowers, has become one of the main components of solid waste in cities [19,20]. In fact, the dry weight of garden waste in Beijing, China, reaches an impressive 3 million tons per year [20], providing ample raw material for biochar production. Utilizing garden waste for biochar production not only mitigates the drawbacks associated with traditional disposal methods like incineration, landfill, and biodegradation but also offers advantages, such as minimal environmental pollution, swift reaction rates, and versatile applications [19,21,22]. Additionally, garden waste exhibits low levels of harmful components, primarily comprising elements like C, O, P, N, K, H, Na, Mg, and Ca [20]. Therefore, the application of garden waste biochar in NO_3_^−^–N adsorption holds significant promise for both wastewater treatment and garden waste management.

The application potential of biochar in contaminant removal is governed by its physical and chemical characteristics, including porosity, specific surface area, and volume, as well as the abundance of functional groups on its surface. These traits are highly influenced by factors such as feedstock, pyrolysis temperature, residence time, and heating rate [15,23,24,25]. Among these factors, pyrolysis temperature has a particularly significant influence on the yield and properties of biochar [26,27]. Studies have shown that high-temperature biochars can effectively remove cations from aqueous solutions and water [26,28]. However, the electrostatic adsorption of biochar with anions, such as NO_3_^−^–N, may be affected by negative charges present on the surface of biochar produced directly from biomass pyrolysis [1,29]. As a result, the anionic sorption characteristics of biochar remain uncertain and are highly dependent on the feedstock and pyrolysis temperature [5]. Additionally, the performance of NO_3_^−^–N removal by biochar is contingent on various adsorption conditions, including initial pH, NO_3_^−^–N concentration, contact time, reaction temperature, and biochar dosage [8,15]. Some studies have reported promising results regarding biochar’s ability to adsorb NO_3_^−^ –N. For instance, biochar derived from pinewood achieved around 95% of nitrate removal with a contact time of 240 min [14]. Mibinuola et al. found that biochar produced from elephant grass exhibited excellent nitrate ion removal, with better adsorption capacity at higher pyrolysis temperatures [16]. Bamboo-derived biochar achieved maximum adsorption of 5 mg/g at pH 4 [30], while maximum values of 0.43, 1.57, and 1.43 mg/g were obtained at pH 7 for 0.2 g biochar produced from *Phragmites communis*, sawdust, and eggshell, respectively [31]. Conversely, some research showed that certain biochars had low or no ability to adsorb NO_3_^−^–N. For instance, Hollister et al. observed no NO_3_^−^–N absorption using biochars from *Zea mays* L. and *Quercus* spp. [32]. The inconsistency among the studies may be due to variations in feedstock, preparation conditions, pH, contact time, initial nitrate concentration, and biochar dosage. Therefore, a comprehensive investigation of the effects of feedstock, preparation, and adsorption conditions on the removal of NO_3_^−^–N is essential to understand and optimize the performance of biochar in this context.

In this study, we sought to explore the NO_3_^−^–N adsorption characteristics of biochar by using eight common garden waste materials as feedstock. The investigation focused on the impact of feedstock type, pyrolysis temperature, initial pH value, initial concentration of NO_3_^−^–N, and biochar dosage on the adsorption process. To gain deeper insights into the adsorption mechanism of NO_3_^−^–N, we applied both the pseudo-first-order and pseudo-second-order kinetic models, as well as the Langmuir, Freundlich, Temkin and Dubinin–Radushkevich models for analysis.

## 2. Materials and Methods

### 2.1. Biochar Preparation and Characterization

In this study, eight different types of garden waste were collected, namely grass clippings (GC), *Rosa chinensis Jacq*. branches (RC), *Prunus persica* branches (PP), *Armeniaca vulgaris Lam.* branches (AV), *Morus alba Linn.* sp. branches (MA), *Platycladus orientalis* (L.) Franco branches (PO), *Pinus tabuliformis Carrière* branches (PT), and *Sophorajaponica Linn*. branches (SL) from the Ecological Restoration Base of the Institute of Resources and Environment, Beijing Academy of Science and Technology (Beijing, China) to produce biochar. The cellulose, hemicellulose, and lignin content of each garden waste type were determined using the normal form washing method. The garden waste samples were thoroughly cleaned with deionized water and dried in the absence of light. GC was pulverized and pressed into cylindrical shapes (approximately 1 cm in diameter and 3 cm in length) using a molding machine. The other garden waste samples were cut into small sections with a cross-section diameter of about 1 cm and a length of 3 to 5 cm. These prepared samples underwent pyrolysis under O-limited conditions, heated from 200 °C to the target temperatures of 300 °C, 500 °C, and 700 °C (with a 60-min hold) at a heating rate of 15 °C/min, using a self-made double-bile cycle rapid cooling waste heat reuse charring furnace. A total of 24 different types of biochars were obtained, categorized as follows: grass biochars (GC300, GC500, GC700), *Rosa chinensis Jacq*. biochars (RC300, RC500, RC700), *Prunus persica* biochars (PP300, PP500, PP700), *Armeniaca vulgaris Lam.* biochars (AV300, AV500, AV700), *Morus alba Linn.* sp. biochars (MA300, MA500, MA700), *Platycladus orientalis* (L.) Franco biochars (PO300, PO500, PO700), *Pinus tabuliformis Carrière* biochars (PT300, PT500, PT700), and *Sophorajaponica Linn*. biochars (SL300, SL500, SL700), produced at three different temperatures. Following production, the biochars were ground into a fine powder, passed through a 200-mesh sieve, and cleaned using 95% alcohol. Subsequently, 20 g of each biochar was mixed with 200 mL of alcohol and shaken at 180 r·min^−1^ at 26 ± 1 °C for 12 h. The solid phase was then separated through suction filtration until the filtrate turned colorless. Finally, the biochar was dried at 65 °C until a constant weight was achieved.

The biochar’s pH was measured using a pH meter (PHS-3C) by mixing the sample with distilled water at a mass:water ratio of 1:20. To determine the ash content, 1.0000 g of biochar sample was burned in a muffle furnace at 750 for 6 h until a constant weight was attained. The crystallographic structure of the biochar was analyzed using X-ray diffraction (XRD) using a D2 PHASER instrument (Bruker Technology Co., Ltd., Deutschland, Germany). Surface functional groups were analyzed via Fourier-transform infrared (FTIR) spectroscopy (NICOLET6700, Thermo Fisher Scientific, Waltham, MA, USA). For the element distribution and surface state analysis, X-ray photoelectron spectroscopy (XPS) was conducted using an Escalab 250Xi instrument (Thermo Fisher Scientific, Waltham, MA, USA). The surface morphology of the biochar was investigated using scanning electron microscopy (SEM; S-4800, Hitachi Ltd., Tokyo, Japan). Lastly, the BET surface area and pore characteristics of the biochar were measured using a Specific surface area and aperture analyzer (ASAP2460, Micromeritics Instrument Corp., Norcross, GA, USA). For the determination of the Zeta potential of biochar, 0.05 g biochar was added into a 50 mL capped glass tube before adding 25 mL 0.01 mol·L^−1^ NaNO_3_ solution. The initial pH of the solution was adjusted to about 2, 4, 6, 8, and 10 by the addition of 1 mol·L^−1^ HNO_3_ or 1 mol·L^−1^ NaOH. After pH adjustment, the mixture was agitated at 120 r·min^−1^ at a temperature of 26 ± 1 °C. Afterward, the Zeta potential was measured with a Malvern analyzer.

### 2.2. Adsorption Experiment

A comprehensive set of NO_3_^−^–N adsorption experiments were conducted in 100 mL polypropylene centrifuge tubes to investigate the effects of various factors on the NO_3_^−^–N adsorption characteristics, including feedstock, pyrolysis temperature, initial pH, contact time, initial concentration, and biochar dosage. The NO_3_^−^–N solutions were prepared using KNO_3_, and the initial pH was adjusted through the addition of HCl and NaOH solutions with a concentration of 1 mol·L^−1^.

#### 2.2.1. Influence of pH

The influence of pH on NO_3_^−^–N adsorption characteristics was studied using 50 mL of 50 mg·L^−1^ NO_3_^−^–N aqueous solutions. The initial pH of NO_3_^−^–N aqueous solution was adjusted to pH 2, 4, 6, 8, 10, and 12, respectively. Then 2.0 g of each biochar was added to the solution, and the mixture was agitated at 180 r·min^−1^ at a temperature of 26 ± 1 °C. After 24 h, the solution was filtered using a needle filter unit with a pore size of 0.45 μm. The concentration of unadsorbed NO_3_^−^–N was detected by a UV spectrophotometer, and the concentration of adsorbed NO_3_^−^–N was calculated as the difference between the initial concentration of NO_3_^−^–N and the concentration of unadsorbed NO_3_^−^–N. To ensure accuracy, three replicates were performed for each treatment.

#### 2.2.2. Influence of Contact Time

To assess the influence of contact time on NO_3_^−^–N adsorption characteristics, 50 mL of 50 mg·L^−1^ NO_3_^−^–N aqueous solutions were prepared. The initial pH of each aqueous solution was adjusted to pH = 2. Then, 2.0 g of each biochar was introduced into the solution. The samples were subjected to agitation at 180 r·min^−1^ and maintained at 26 ± 1 °C for varying durations of 1 h, 3 h, 6 h, 12 h, 24 h, and 48 h. Subsequently, the solution was filtered, and the concentration of unadsorbed NO_3_^−^–N was determined following the methods described in Section 2.2.1.

#### 2.2.3. Influence of Initial Concentration of NO_3_^−^–N

The influence of the initial concentration of NO_3_^−^–N on its adsorption was investigated using 50 mL of NO_3_^−^–N aqueous solutions with concentrations of 5, 10, 20, 50, 100, 200, and 400 mg·L^−1^. Similar to previous steps, the initial pH of NO_3_^−^–N aqueous solution was adjusted to pH = 2, and 2.0 g of each biochar was added to the mixture. Each mixture was shaken at 180 r·min^−1^ and kept at 26 ± 1 °C for 24 h. Afterward, the solution was filtered, and the concentration of unadsorbed NO_3_^−^–N was determined according to the methods described in Section 2.2.1.

#### 2.2.4. Influence of Biochar Dosage

To investigate the influence of dosage on NO_3_^−^–N adsorption characteristics, experiments were conducted using 50 mL of 50 mg·L^−1^ NO_3_^−^–N aqueous solutions. The initial pH of each NO_3_^−^–N aqueous solution was adjusted to pH = 2. Then varying amounts of biochar (1.0, 1.5, 2.0, 2.5, and 3.0 g) were added to individual solutions. After thorough shaking, each mixture was filtered, and the concentration of unadsorbed NO_3_^−^–N was determined using the methods described in Section 2.2.1.

### 2.3. Calculation and Statistical Methods

The biochar yield (*W*, %) at each temperature was calculated using the following equation:

(1) $$W=\frac{m_{2}}{m_{1}}\times 100\%$$

where *m*_1_ and *m*_2_ (g) represent the mass of the feedstock and biochar, respectively.

The ash content (*A*, %) of the biochar was calculated as follows:

(2) $$A=\frac{m_{4}}{m_{3}}\times 100\%$$

where *m*_3_ and *m*_4_ (g) are the mass of the biochar sample before and after burning, respectively.

The removal efficiency (*R*, %) and adsorption capacity (*q*, mg·g^−1^) of the biochar on NO_3_^−^–N were calculated using the following equations:

(3) $$R=\frac{C_{0}-C_{t}}{C_{0}}\times 100\%$$

(4) $$q=\frac{(C_{0}-C_{t})\times V}{M}$$

where *C*_0_ and *C_t_* (mg·L^−1^) are the initial and time *t* concentrations of NO_3_^−^–N, respectively, *V* (L) is the volume of the adsorption solution, and *M* (g) is the amount of biochar added.

For the kinetic analysis of NO_3_^−^–N adsorption, pseudo-first-order and pseudo-second-order kinetic models were employed:

(5) $$\ln(q_{e}-q_{t})=lnq_{e}-k_{1}t$$

(6) $$\frac{t}{q_{t}}=\frac{1}{k_{2}q_{e}^{2}}+\frac{t}{q_{e}}$$

where *q_e_* (mg·g^−1^) is the adsorption capacity at equilibrium; *t* (min) is the adsorption time, *q_t_* (mg·g^−1^) is the adsorption amount at time *t*, *k*_1_ (min^−1^) and *k*_2_ (mg·g^−1^·min^−1^) are the constants for pseudo-first-order and pseudo-second-order kinetics models, respectively.

Various isotherm models, including Langmuir, Freundlich, Temkin, and Dubinin–Radushkevich models, were used for the isotherm analysis:

(7) $$\frac{C_{e}}{q_{e}}=\frac{C_{e}}{q_{max}}+\frac{1}{q_{max}K_{L}}$$

(8) $$lnq_{e}=lnK_{F}+\frac{1}{n}lnC_{e}$$

(9) $$q_{e}=a+blnC_{e}$$

(10) $$q_{e}=q_{max}\exp\left\{-\beta {\left[RT\ln\left(1+\frac{1}{C_{e}}\right)\right]}^{2}\right\}$$

where *C_e_* (mg·L^−1^) is the concentration of NO_3_^−^–N at equilibrium, *q_max_* (mg·g^−1^) is the theoretical maximum adsorption capacity, *K_L_* (L·mg^−1^) and *K_F_* are the constants for the Langmuir and Freundlich isotherm models, respectively, *n* is the Freundlich index, *a* and *b* are constants related to the energy and capacity of adsorption, respectively, *β* is the activity coefficient related to the mean adsorption energy per mole (mol^2^·kJ^−2^), *R* is the universal gas constant (8.314 kJ·K^−1^·mol^−1^), and *T* is the absolute temperature (K).

## 3. Results and Discussion

### 3.1. Biochar Characteristics

#### 3.1.1. Yield, pH and Ash

The biochar yields, pH and ash content were influenced by both the pyrolysis temperature and the type of garden waste used as feedstock (Table 1). Notably, the biochar yields exhibited a significant decrease with increasing pyrolysis temperature, while the pH and ash content showed an opposite trend (*p* < 0.05). These findings are consistent with previous research conducted by other scholars [5,31].

At the pyrolysis temperature of 300 °C, 500 °C, and 700 °C, the biochar yields ranged from 25.26% to 47.09%, 19.14% to 35.57%, and 14.43% to 27.01%, respectively. Among the different garden waste types, AV showed the highest yields at each pyrolysis temperature. These differences in biochar yields can primarily be explained by condensation polymerization and more pronounced destruction of macromolecular constituents at higher pyrolysis temperatures [33]. As the temperature increases, the dehydration of hydroxyl groups and thermal degradation of biomass components, such as cellulose, hemicellulose, and lignin, become more pronounced. Hemicellulose tends to decompose most easily in the temperature range of 200–300 °C, followed by cellulose in the range of 300–380 °C and lignin in the range of 200–500 °C [14,17,34]. Additionally, as shown in Table 2, the content of cellulose, hemicellulose, and lignin significantly varied with the feedstock (*p* < 0.05), resulting in different biochar yields for each type of garden waste at a given pyrolysis temperature. For instance, the biochar yield of GC experienced a slight decrease with increasing pyrolysis temperature, likely because hemicellulose, with the highest content, had already decomposed before reaching 300 °C. In contrast, the slight decrease in PO’s biochar yield can be attributed to its highest lignin content, which remains more stable at higher pyrolysis temperatures.

The pH values of all biochars were found to be greater than 7. This rise in pH with increasing pyrolysis temperature can be attributed to the removal of acidic functional sites and the potential increase in alkaline concentrations or mineral contents within the biochar [35]. As the pyrolysis temperature increases, more organic components in the garden waste are pyrolyzed, leading to a substantial increase in ash content [17]. Moreover, variations in pH and ash content were observed among different types of garden waste at a given pyrolysis temperature, highlighting the influence of the feedstock on these characteristics.

#### 3.1.2. XRD, FTIR and XPS Analysis

To explore the crystallinity of the biochar samples, XRD patterns were obtained and are presented in Figure S1. Each biochar exhibited a typical characteristic peak (002) of graphitic carbon, indicating its amorphous state [1]. Furthermore, the XRD analysis also revealed the presence of Na and Ca elements in the biochar samples. Notably, the pyrolysis temperature influenced the forms of Ca crystallization peaks observed in the biochar. Specifically, a Ca crystallization peak was observed at the pyrolysis temperature of 300 °C, while CaCO_3_ and CaO were detected at the pyrolysis temperatures of 500 °C and 700 °C, respectively.

Additional evidence concerning the effect of pyrolysis temperature and garden waste type on the surface binding sites of biochar was obtained through FTIR analyses (Figure S2). The total functional groups of the biochars gradually decreased as the pyrolysis temperature increased [17], with variations in their extent/presence observed among the different biochars due to the diverse garden waste types. Specifically, the –OH stretching vibration (3650–3400 cm^−1^) decreased with increasing pyrolysis temperature, which can be explained by the dehydration of the feedstock [14,25]. Moreover, the C≡N stretching (2500–2300 cm^−1^) of biochar samples produced from GC and RC disappeared entirely at the pyrolysis temperature of 700 °C. Similarly, the C=O stretching (1750–1650 cm^−1^) in aldehydes, ketone groups, and esters, as well as the C=C stretching (1600–1520 cm^−1^) in aromatic skeletal bands, the C-O-C stretching (1300–1100 cm^−1^) in vibration in esters and anhydrides, and the aromatic C–H out-of-plane bending vibrations (870–800 cm^−1^) declined as the pyrolysis temperature increased. These findings indicate biopolymer decomposition at higher pyrolysis temperatures. The wide-scale XPS spectra revealed the presence of C, O, N, and Ca elements with binding energies at 292.28, 539.07, 405.01, and 354.47 eV, respectively (Figure S3). These peaks were attributed, respectively, to C1s, O1s, N1s, and Ca2p. In addition, C and O were the predominant elements in each biochar sample, which is consistent with findings reported by other researchers [1,27].

#### 3.1.3. SEM and BET Analysis

Scanning electron micrographs of the biochars are provided in Figure S4. As expected, the pore shapes, sizes, and distributions were different among the biochar samples, which could be explained by the different garden waste types and pyrolysis temperatures. Generally, the porosity and pore volumes increased with increasing pyrolysis temperatures, and a honeycomb-like structure was observed in most biochar samples at higher pyrolysis temperatures. Similar results were reported by other researchers in this regard [14,31]. However, no honeycomb-like structure was displayed in biochars GC500, GC700, RC500, RC700, AV500, and AV700.

In line with the SEM results, the total pore volume of biochars derived from PP, AV, MA, and PO increased with rising pyrolysis temperature, whereas the opposite trend was observed for RC (Table 3). Moreover, the BET surface area of biochars produced from GC and RC decreased with increasing pyrolysis temperature (*p* < 0.05), which can be attributed to the destruction of the pore structure. Hollister et al. also reported a higher specific surface area at lower pyrolysis temperatures [32]. However, contrary results have been demonstrated by several other researchers [5,14,27,31]. For instance, at pyrolysis temperatures of 300 °C, 500 °C, and 700 °C, the BET surface areas were 185.232 (GC300), 9.418 (PP500), and 270.801 m^2^·g^−1^ (PO700), respectively. In addition, the average pore diameters were significantly influenced by pyrolysis temperature and garden waste type (*p* < 0.05).

### 3.2. Influence of pH

**Figure 1:**
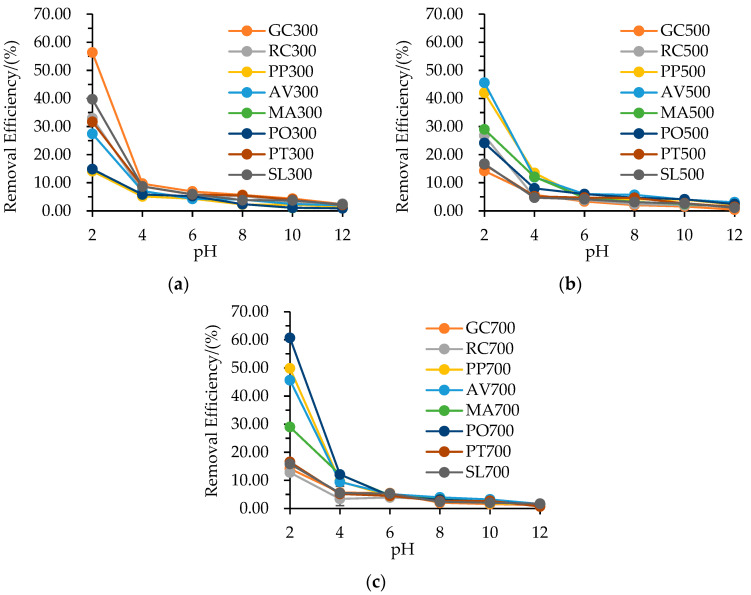
Effect of initial pH on NO_3_^−^–N adsorption by different garden waste biochars at different pyrolysis temperatures: (**a**) 300 °C, (**b**) 500 °C, (**c**) 700 °C.

The pH value is one of the most important factors affecting the adsorption process [1,15]. Figure 1 illustrates the effect of the initial pH of the NO_3_^−^–N solution on its adsorption by the biochars. The removal efficiency of NO_3_^−^–N decreased as the initial pH value increased from 2 to 12 for each biochar at a given pyrolysis temperature, with a more substantial decrease observed when the pH value increased from 2 to 4. At pH 2, the maximum removal efficiencies were 56.33% (GC300), 45.59% (AV500) and 60.67% (PO700) under pyrolysis temperatures of 300 °C, 500 °C and 700 °C, respectively. On the contrary, the minimum values were 0.93% (PO300), 0.42% (GC500) and 0.72% (PT700) at pH 12, respectively. The pH not only affected the surface charges and dissociation of functional groups on the biochar but also influenced the chemical speciation and diffusion rate of nitrate [29,36]. At lower pH values, the presence of H^+^ led to an increase in the number of positively charged sites. Consequently, the active sites on the biochar’s surface favored the adsorption of NO_3_^-^ through electrostatic interactions. Conversely, at relatively higher pH values, the abundance of OH^-^ would hinder the adsorption capacity [1,29,37]. Moreover, results of the Zeta potential of different biochars in this study (Figure S5) showed that the surface of each biochar was positively charged at pH 2, and biochar could adsorb nitrate due to coulombic attraction. While at pH ≥ 4, the surface of biochar was negatively charged, resulting in the decrease of NO_3_^−^–N removal.

The removal efficiency of NO_3_^−^–N also showed variations among the different garden waste biochars at a given pH value and pyrolysis temperature, especially at pH 2. These differences could be explained by the distinct specific surface areas and pore volumes of the biochars [1,15]. For instance, GC300 and PO700 displayed higher removal efficiency because of their larger specific surface areas of 185.232 and 270.801 m^2^·g^−1^ and total pore volumes of 0.122 and 0.155 m^3^·g^−1^, respectively (Table 3). Furthermore, the specific surface area and total pore volume of GC, RC, and SL biochars generally decreased with increasing pyrolysis temperature, whereas the opposite trend was observed for PP, AV, and PO biochars. This pattern can explain the similar trend observed in their removal efficiency of NO_3_^−^–N with increasing pyrolysis temperature at a given pH.

### 3.3. Influence of Contact Time

**Figure 2:**
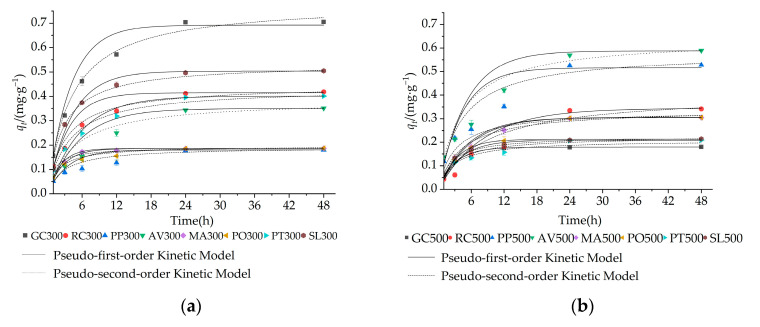

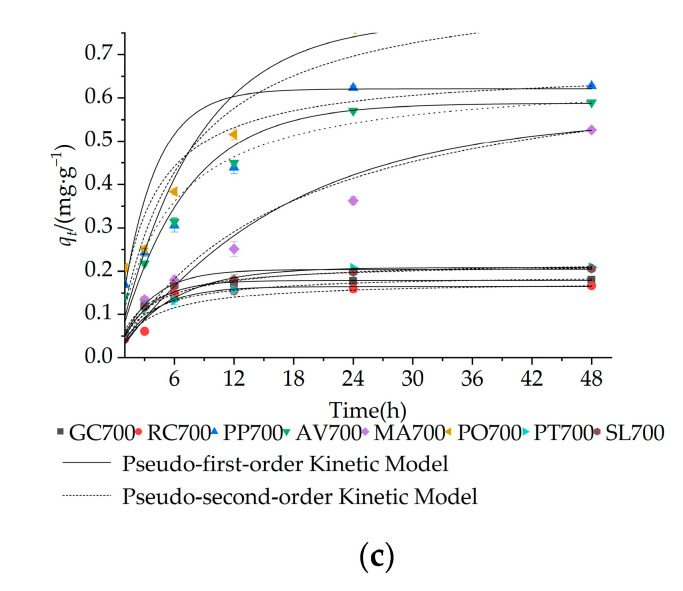
Effect of contact time on NO_3_^−^–N adsorption and kinetics fitting results of different garden waste biochars at different pyrolysis temperatures: (**a**) 300 °C, (**b**) 500 °C, (**c**) 700 °C.

Contact time is a key factor in evaluating the efficiency of biochar, as it primarily depends on the reaction mechanism [30,38]. In line with findings from other researchers, the adsorption amount of each biochar increased with increasing contact time until it reached a relative equilibrium state [17,39] (Figure 2). The removal of NO_3_^−^–N was affected by the garden waste type and pyrolysis temperature, resulting in varying adsorption amounts of NO_3_^−^–N at a certain contact time. The equilibrium adsorption amount of NO_3_^−^–N ranged from 0.17 (RC700) to 0.78 mg·g^−1^ (PO700). Moreover, the equilibrium adsorption amount of NO_3_^−^–N for GC, RC and SL biochars decreased as the pyrolysis temperature increased, whereas the opposite trend was observed for PP, AV, MA and PO biochars, This can be explained by the variations in their specific surface area with pyrolysis temperature (Table 3). During the initial 24 h of the reaction, rapid adsorption occurred, after which the process reached equilibrium. This rapid removal rate of NO_3_^−^–N at the beginning can be explained by the higher initial NO_3_^−^–N concentration and the larger number of active sites on the biochar surface during the early stages of the reaction [30]. As the adsorption process continued, the active sites gradually diminished, leading to a balanced reaction [1,40].

In this study, pseudo-first-order and pseudo-second-order kinetic models were used to analyze the kinetic mechanism of NO_3_^−^–N adsorption (Figure 2 and Table 4). The correlation coefficients (*R*^2^) for both models were higher than 0.900, indicating a good fit. The results suggested that chemical adsorption likely served as the rate-controlling mechanism for NO_3_^−^–N removal at the pyrolysis temperature of 300 °C (except for PT300 and SL300) and 700 °C (except for GC700), as evidenced by the generally higher *R*^2^ values for the pseudo-second-order kinetic model [39]. At the pyrolysis temperature of 500 °C, the pseudo-first-order kinetic model was more suitable for describing the NO_3_^−^–N adsorption behavior in GC500, RC500, MA500, and PO500, as it yielded higher *R*^2^ values compared to the pseudo-second-order kinetic model, indicating a physical adsorption rate mechanism for NO_3_^−^–N removal [17,41]. In contrast, PP500, AV500, PO500 and PT500 showed a chemical adsorption process. Hence, it is evident that the removal mechanism of NO_3_^−^–N was affected by both the feedstock and pyrolysis temperature.

### 3.4. Influence of Initial Concentration of NO_3_^−^–N

**Figure 3:**
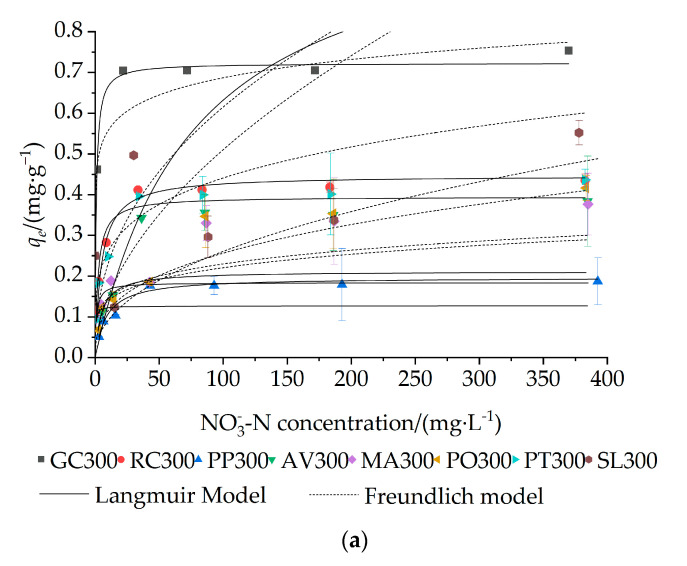

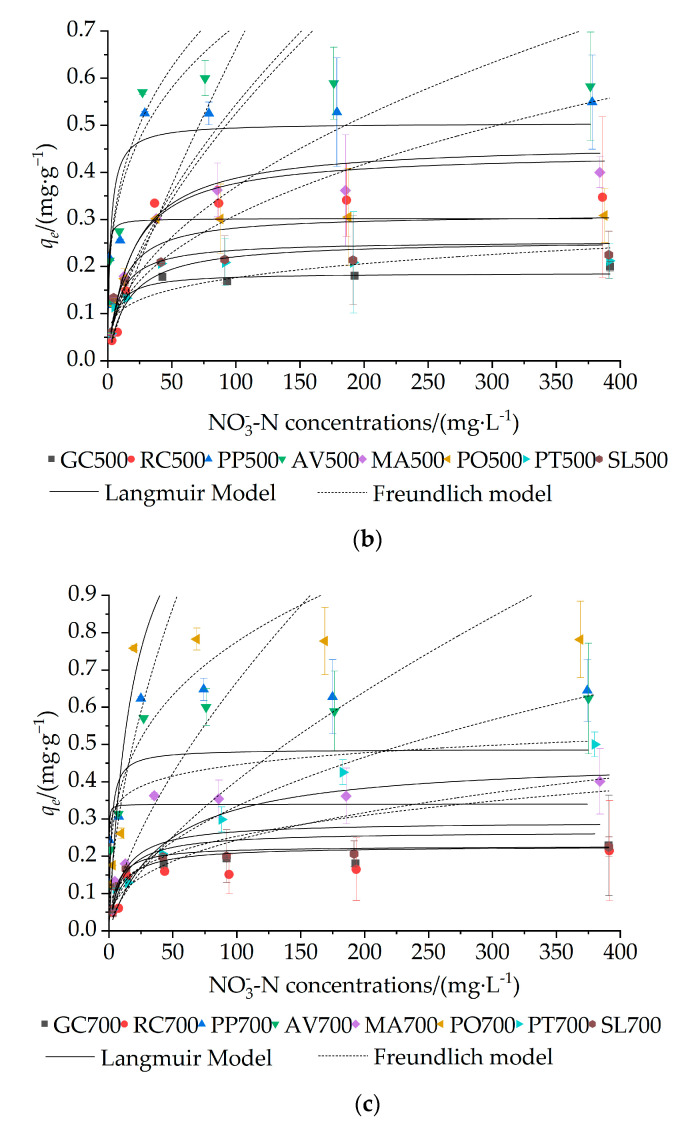
Effect of NO_3_^−^–N concentration on adsorption and isotherm fitting results of different garden waste biochars at different pyrolysis temperatures: (**a**) 300 °C, (**b**) 500 °C, (**c**) 700 °C.

The initial concentration of NO_3_^−^–N is another important factor influencing the adsorption of NO_3_^−^–N by biochar. As depicted in Figure 3, the adsorption capacity of NO_3_^−^–N tended to increase with the increasing initial concentration of NO_3_^−^–N for the same amount of biochar, a phenomenon also reported by other researchers [5,30]. The adsorption capacity of each biochar exhibited rapid increase with increasing NO_3_^−^–N concentration from 5 to 50 mg·L^−1^, followed by a slower increase until it reached a relative equilibrium state. This behavior can be attributed to the higher driving force at higher concentrations, which led to the rapid occupation of adsorption sites [27]. Consequently, the adsorption capacity stabilized as the concentration of NO_3_^−^–N continued to increase. Under the equilibrium state at a NO_3_^−^–N concentration of 400 mg·L^−1^, the order of NO_3_^−^–N adsorption capacity was as follows: GC300 > SL300 > PT300 > RC300 > PO300 > AV300 = MA300 > PP300, AV500 > PP500 > MA500 > RC500 > PO500 > SL500 > PT500 > GC500, and PO700 > PP700 > AV700 > PT500 > MA700 > SL700 = GC700 > RC700, at the pyrolysis temperature of 300 °C, 500 °C, and 700 °C, respectively. These results indicated that the NO_3_^−^–N adsorption capacity on biochars was also affected by the pyrolysis temperature and garden waste type [31].

The adsorption capacity data were modeled using Langmuir, Freundlich, Temkin and Dubinin–Radushkevich models for the isotherm analysis (Figure 3 and Table 5). The Freundlich model showed a better fit to the adsorption data of RC300, MA300, PP500, and AV700 (0.972 > *R*^2^ > 0.903) than the Langmuir model (0.954 > *R*^2^ > 0.795), indicating a heterogeneous surface of biochar [20]. The values of 1/n obtained from the Freundlich model ranged from 0.198 to 0.297, suggesting a favorable adsorption process [20]. On the other hand, the Langmuir model provided a better fit to the adsorption data of GC300, PP300, PO300, PT300, MA500, PO500, PT500, PO700, PT700, and SL700 (0.999 > *R*^2^ > 0.925) compared to the Freundlich model (0.996 > *R*^2^ > 0.751), assuming that the adsorption of NO_3_^−^–N followed typical monolayer adsorption with uniform adsorption positions [20]. Therefore, the Langmuir model was deemed more suitable to describe more of the adsorption capacities of biochars in this study, which is consistent with the results found by other researchers investigating nitrate removal [8,17]. According to the Langmuir model, the maximum adsorption capacities of NO_3_^−^–N were determined to be 0.723 (GC300) and 1.339 mg·g^−1^ (PO700) at the pyrolysis temperature of 300 and 700 °C, respectively, which can be explained by the relative greater specific surface areas and pore volumes of the biochars at a given pyrolysis temperature (Table 3). 

It can be noticed that the Temkin model exhibited a good fit to the experimental data of RC300, MA300, PO300, PT300, MA500, and PT700 (0.955 > *R*^2^ > 0.901). Moreover, the positive b values obtained from this model indicated an exothermic process of NO_3_^−^–N adsorption [8], suggesting the presence of electrostatic interactions [42].

The Dubinin–Radushkevich model exhibited a good fit to the experimental data of GC300, GC500, SL500, GC700, and SL700 (0.980 > *R*^2^ > 0.941). Moreover, this model provides mean adsorption energy (E, kJ·mol^−1^) that is equal to $1/\sqrt{2\beta }$. This parameter is valuable in predicting the type of adsorption. If the E value is less than 8 kJ·mol^−1^, it indicates physical adsorption, while a range of 8–16 kJ·mol^−1^ signifies chemical adsorption [30,43]. In Table 5, it can be observed that all the E values of biochar samples are less than 8 kJ·mol^−1^, indicating a physical nature of adsorption.

### 3.5. Influence of Biochar Dosage

**Figure 4:**
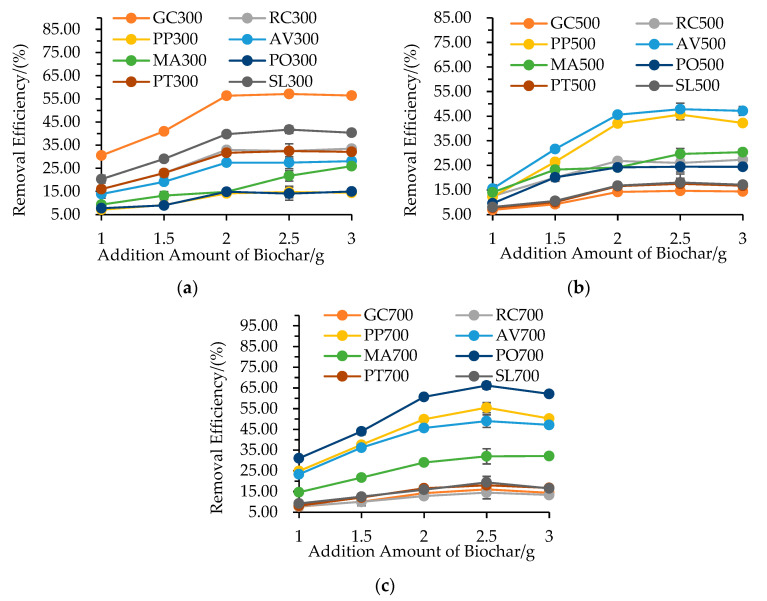
Effect of biochar dosage on NO_3_^−^–N concentration by different garden waste biochars at different pyrolysis temperatures: (**a**) 300 °C, (**b**) 500 °C, (**c**) 700 °C.

As shown in Figure 4, the NO_3_^−^–N removal efficiency exhibited an initial increase followed by a plateau as the biochar dosage increased, which aligns with the studies involving NO_3_^−^–N adsorption [8,29]. This behavior can be explained by the availability of more active sites on the biochar surfaces, facilitating NO_3_^−^–N adsorption [43]. Notably, a significant increase in NO_3_^−^–N removal efficiency was observed as the biochar dosage increased from 1.0 to 2.0 g. The removal efficiency also showed variations among the different garden waste biochars at a given biochar dosage and pyrolysis temperature. Moreover, the maximum NO_3_^−^–N removal efficiency values were observed for GC300, AV500 and PO700 at the pyrolysis temperature of 300 °C, 500 °C and 700 °C, respectively. These differences can be attributed to variations in specific surface area and pore volume among the biochars due to differences in pyrolysis temperature and garden waste type [1,15].

## 4. Conclusions

Biochar characteristics were significantly influenced by garden waste type and pyrolysis temperature. The biochar yields exhibited a decreasing trend with increasing pyrolysis temperature, while the pH value and ash content increased as the pyrolysis temperature increased. The NO_3_^−^–N removal process was found to be highly dependent on various factors, including feedstock, pyrolysis temperature, initial pH and initial concentration of NO_3_^−^–N, contact time, and biochar dosage. Notably, each type of garden waste biochar demonstrated effective NO_3_^−^–N removal under acidic conditions, with maximum removal efficiency observed at pH 2. Furthermore, contact time, initial concentration of NO_3_^−^–N, and biochar dosage exerted positive effects on NO_3_^−^–N removal. The pseudo-second-order kinetic model fitted the most adsorption capacity data of biochars. The maximum NO_3_^−^–N adsorption capacity was observed in PO700, reaching 1.339 mg·g^−1^. The safety of the biochars produced from garden waste, such as the presence of heavy metals, should be considered in further studies.

## Figures and Tables

**Table 1:** Biochar yield, pH, and ash content at different pyrolysis temperatures for different garden wastes.

Samples	Yield (%)	pH	Ash (%)
GC300	34.89 ± 0.11 j	7.26 ± 0.06 abc	11.15 ± 0.17 n
GC500	31.95 ± 0.87 ef	7.84 ± 0.05 f	22.29 ± 0.02 o
GC700	25.81 ± 1.42 j	10.03 ± 0.10 k	27.93 ± 0.03 p
RC300	25.56 ± 0.54 l	8.04 ± 0.07 g	4.40 ± 0.16 d
RC500	19.99 ± 0.94 j	8.37 ± 0.17 h	6.15 ± 0.12 h
RC700	16.51 ± 0.66 i	8.52 ± 0.11 hi	6.25 ± 0.10 h
PP300	35.67 ± 1.53 f	7.25 ± 0.06 abc	4.24 ± 0.12 cd
PP500	28.87 ± 0.75 k	8.03 ± 0.04 g	5.90 ± 0.21 g
PP700	26.48 ± 0.46 i	9.32 ± 0.04 j	6.78 ± 0.07 j
AV300	47.09 ± 0.23 cd	7.48 ± 0.07 de	4.29 ± 0.17 d
AV500	35.57 ± 0.70 h	7.92 ± 0.14 fg	6.14 ± 0.03 h
AV700	27.01 ± 0.46 j	8.64 ± 0.07 i	6.66 ± 0.06 j
MA300	34.69 ± 0.69 i	7.20 ± 0.06 ab	4.34 ± 0.03 d
MA500	32.13 ± 2.68 f	8.06 ± 0.09 g	5.65 ± 0.06 f
MA700	21.26 ± 1.10 c	9.93 ± 0.10 k	7.26 ± 0.03 k
PO300	32.65 ± 0.04 gh	7.22 ± 0.07 ab	3.06 ± 0.05 a
PO500	25.97 ± 0.44 f	7.50 ± 0.08 e	3.19 ± 0.04 ab
PO700	25.21 ± 0.37 b	8.38 ± 0.06 h	3.25 ± 0.07 b
PT300	26.40 ± 0.39 f	7.11 ± 0.09 a	4.10 ± 0.10 c
PT500	19.14 ± 1.39 fg	7.42 ± 0.09 cde	5.37 ± 0.02 e
PT700	14.43 ± 0.52 d	10.03 ± 0.13 k	7.89 ± 0.09 m
SL300	39.47 ± 0.80 ef	7.30 ± 0.05 bcd	4.26 ± 0.04 cd
SL500	28.39 ± 0.54 a	7.47 ± 0.26 de	6.45 ± 0.12 i
SL700	23.86 ± 2.14 e	9.97 ± 0.10 k	7.45 ± 0.06 l

Data represent means ± SD of n = 3. Different lowercase letters within the same column indicate significant variations (*p* < 0.05) in yield, pH, or ash content among the samples.

**Table 2:** Cellulose, hemicellulose and lignin content in selected garden waste.

Samples	Cellulose (%)	Hemicellulose (%)	Lignin (%)
GC	19.30 ± 0.52 a	24.61 ± 0.70 d	2.18 ± 0.31 a
RC	35.44 ± 0.65 b	15.66 ± 0.81 ab	13.81 ± 0.14 b
PP	43.79 ± 1.40 d	23.55 ± 2.48 d	16.62 ± 0.27 c
AV	38.70 ± 0.86 c	20.15 ± 1.30 c	22.11 ± 0.71 d
MA	40.05 ± 3.19 c	19.83 ± 3.17 c	15.72 ± 0.63 c
PO	44.73 ± 0.39 d	14.98 ± 1.46 a	27.88 ± 1.18 e
PT	34.69 ± 0.52 b	14.59 ± 0.17 a	22.89 ± 0.33 d
SL	34.65 ± 1.31 b	18.47 ± 0.54 bc	16.29 ± 0.63 c

Data represent means ± SD of n = 3. Different lowercase letters in the same column indicate significant variations (*p* < 0.05) in cellulose, hemicellulose, or lignin content among the samples.

**Table 3:** Specific surface area, total pore volume, and pore size of different biochars.

Samples	Specific Surface Area/(m^2^·g^−1^)	Total Pore Volume/(cm^3^·g^−1^)	Average Pore Diameter/(nm)
GC300	185.232 ± 13.544 g	0.122 ± 0.013 d	2.590 ± 0.568 a
RC300	36.772 ± 0.759 e	0.032 ± 0.005 c	3.018 ± 0.113 a
PP300	2.616 ± 0.067 ab	0.007 ± 0.002 a	12.721 ± 0.917 efg
AV300	1.527 ± 0.090 a	0.005 ± 0.001 a	14.678 ± 0.725 g
MA300	2.300 ± 0.126 ab	0.006 ± 0.002 a	12.827 ± 0.627 efg
PO300	1.310 ± 0.075 a	0.005 ± 0.001 a	13.047 ± 0.942 efg
PT300	1.182 ± 0.148 a	0.004 ± 0.001 a	12.652 ± 0.496 defg
SL300	21.549 ± 2.100 d	0.018 ± 0.004 b	3.300 ± 0.216 a
GC500	2.285 ± 0.206 ab	0.005 ± 0.001 a	12.474 ± 1.000 def
RC500	1.073 ± 0.029 a	0.006 ± 0.001 a	22.216 ± 0.484 i
PP500	9.418 ± 1.164 c	0.020 ± 0.005 b	8.144 ± 0.239 b
AV500	8.624 ± 1.514 bc	0.020 ± 0.011 b	9.636 ± 2.776 bc
MA500	1.774 ± 0.075 a	0.006 ± 0.002 a	17.398 ± 1.504 h
PO500	2.460 ± 0.165	0.008 ± 0.001 a	12.686 ± 0.059 defg
PT500	3.179 ± 0.270 ab	0.009 ± 0.002 a	10.698 ± 0.928 cd
SL500	2.955 ± 0.115 ab	0.007 ± 0.002 a	9.648 ± 0.169 bc
GC700	1.869 ± 0.066 a	0.008 ± 0.001 a	13.370 ± 1.610 fg
RC700	0.465 ± 0.032 a	0.005 ± 0.001 a	46.413 ± 1.715 k
PP700	51.207 ± 1.710 f	0.039 ± 0.002 c	3.217 ± 0.152 a
AV700	10.924 ± 0.197 c	0.031 ± 0.002 c	11.082 ± 0.978 cde
MA700	1.703 ± 0.120 a	0.013 ± 0.002 ab	25.454 ± 0.651 j
PO700	270.801 ± 9.214 h	0.155 ± 0.013 e	2.254 ± 0.291 a
PT700	0.831 ± 0.137 a	0.006 ± 0.001 a	24.071 ± 1.108 j
SL700	1.297 ± 0.186 a	0.008 ± 0.001 a	22.079 ± 2.216 i

Data represent means ± SD of n = 3. Different lowercase letters within the same column indicate significant variations (*p* < 0.05) in specific surface area, total pore volume, or average pore diameter among the samples.

**Table 4:** Kinetic parameters for NO_3_^−^–N adsorption onto different garden waste biochars.

Samples	Pseudo-First-Order Kinetic Model			Pseudo-Second-Order Kinetic Model		
	*q_e_* (mg·g^−1^)	*k*_1_ (min^−1^)	*R* ^2^	*q_e_* (mg·g^−1^)	*k*_2_ (mg·g^−1^·min^−1^)	*R* ^2^
GC300	0.692	0.253	0.994	0.784	0.315	0.998
RC300	0.415	0.285	0.986	0.445	0.725	0.996
PP300	0.180	0.286	0.985	0.191	1.881	0.992
AV300	0.349	0.180	0.961	0.386	0.568	0.970
MA300	0.186	0.445	0.913	0.191	4.804	0.962
PO300	0.186	0.374	0.978	0.196	2.804	0.991
PT300	0.401	0.175	0.983	0.434	0.623	0.971
SL300	0.503	0.238	0.991	0.534	0.705	0.986
GC500	0.179	0.321	0.999	0.208	1.751	0.980
RC500	0.344	0.120	0.996	0.407	0.292	0.994
PP500	0.516	0.228	0.976	0.581	0.418	0.988
AV500	0.588	0.196	0.986	0.641	0.376	0.994
MA500	0.305	0.186	0.999	0.340	0.699	0.987
PO500	0.306	0.174	0.992	0.333	1.002	0.927
PT500	0.208	0.256	0.983	0.222	1.590	0.988
SL500	0.213	0.297	0.988	0.225	1.955	0.994
GC700	0.179	0.320	0.999	0.193	1.832	0.996
RC700	0.165	0.276	0.966	0.178	1.715	0.977
PP700	0.621	0.296	0.982	0.668	0.486	0.992
AV700	0.588	0.149	0.979	0.651	0.317	0.989
MA700	0.560	0.058	0.988	0.719	0.079	0.992
PO700	0.778	0.127	0.970	0.880	0.180	0.976
PT700	0.209	0.182	0.945	0.224	1.436	0.963
SL700	0.204	0.302	0.985	0.218	1.810	0.998

**Table 5:** Langmuir, Freundlich, Temkin and Dubinin–Radushkevich parameters for different garden waste biochars.

Samples	Langmuir			Freundlich			Temkin			Dubinin–Radushkevich			
	*q_max_* (mg·g^−1^)	*k_L_* (L·mg^−1^)	*R* ^2^	*k_F_* (mg·g^−1^)	1/*n* (mg·g^−1^·min^−1^)	*R* ^2^	*a*	*b*	*R* ^2^	*q_max_* (mg·g^−1^)	*Β* (mol^2^·kJ^−2^)	*R* ^2^	*E* (kJ·mol^−1^)
GC300	0.723	1.139	0.988	0.451	0.091	0.966	0.477	0.050	0.849	0.717	0.287	0.980	1.320
RC300	0.395	0.439	0.906	0.153	0.230	0.966	0.163	0.052	0.930	0.353	0.153	0.726	1.809
PP300	0.197	0.118	0.961	0.031	0.459	0.950	0.033	0.029	0.888	0.157	2.303	0.803	0.466
AV300	0.127	2.770	0.243	0.087	0.201	0.461	0.061	0.058	0.857	0.263	0.412	0.422	1.101
MA300	0.184	0.937	0.954	0.092	0.198	0.972	0.063	0.051	0.901	0.246	0.408	0.503	1.107
PO300	0.211	0.210	0.967	0.052	0.349	0.935	0.008	0.069	0.923	0.256	1.738	0.634	0.536
PT300	0.447	0.208	0.947	0.085	0.429	0.765	0.116	0.060	0.920	0.361	0.691	0.876	0.851
SL300	0.466	0.065	0.341	0.042	0.043	0.538	0.022	0.078	0.359	0.432	44.930	0.690	0.105
GC500	0.187	0.188	0.894	0.063	0.223	0.734	0.059	0.025	0.810	0.178	2.527	0.961	0.445
RC500	0.256	0.063	0.655	0.015	0.828	0.892	0.035	0.074	0.840	0.256	4.590	0.681	0.330
PP500	0.302	2.442	0.883	0.181	0.297	0.903	0.203	0.065	0.886	0.424	0.087	0.686	2.395
AV500	0.504	0.464	0.737	0.183	0.313	0.887	0.195	0.078	0.795	0.510	0.551	0.614	0.953
MA500	0.463	0.051	0.926	0.031	0.615	0.851	0.013	0.070	0.955	0.313	3.021	0.890	0.407
PO500	0.443	0.056	0.999	0.033	0.611	0.996	0.037	0.053	0.865	0.266	2.635	0.876	0.436
PT500	0.254	0.120	0.957	0.042	0.432	0.843	0.053	0.031	0.864	0.189	1.962	0.887	0.505
SL500	0.311	0.095	0.768	0.039	0.490	0.569	0.074	0.029	0.825	0.208	1.870	0.977	0.517
GC700	0.228	0.111	0.864	0.042	0.382	0.682	0.050	0.030	0.861	0.188	2.682	0.941	0.432
RC700	0.463	0.024	0.727	0.018	0.671	0.600	0.027	0.030	0.830	0.151	2.280	0.653	0.468
PP700	0.340	10.138	0.499	0.280	0.101	0.617	0.312	0.063	0.795	0.550	0.051	0.567	3.131
AV700	0.487	0.612	0.795	0.200	0.294	0.948	0.216	0.077	0.873	0.532	0.447	0.702	1.058
MA700	0.293	0.098	0.688	0.032	0.659	0.630	0.022	0.070	0.890	0.323	3.104	0.892	0.401
PO700	1.339	0.051	0.937	0.097	0.560	0.751	0.107	0.134	0.719	0.661	2.666	0.689	0.433
PT700	0.267	0.106	0.925	0.037	0.480	0.851	0.064	0.088	0.934	0.270	2.664	0.644	0.433
SL700	0.228	0.186	0.955	0.067	0.288	0.831	0.063	0.030	0.865	0.198	2.005	0.962	0.499

## Data Availability

The data presented in this study are available on request from the corresponding author.

## Supplementary Materials

The following supporting information can be downloaded at: https://www.mdpi.com/article/10.3390/ma16165726/s1, Figure S1: XRD patterns of different biochars; Figure S2: FTIR spectra of different biochars; Figure S3: The wide-scale XPS spectra of different biochars; Figure S4: SEM of different biochars; Figure S5: Zeta potential of different biochars.

## References

[1] Wang W.X., Zhu Q., Huang R.Y., Hu Y.H. (2023). Adsorption of nitrate in water by CTAB-modified MgFe layered double hydroxide composite biochar at low temperature: Adsorption characteristics and mechanisms. J. Environ. Chem. Eng..

[2] Chen Y.T., Yan J., Chen M.L., Guo F.C., Liu T., Chen Y. (2022). Effect of wetland plant fermentation broth on nitrogen removal and bioenergy generation in constructed wetland-microbial fuel cells. Front. Environ. Sci. Eng..

[3] Guo F.C., Luo Y., Nie W.B., Xiong Z.C., Yang X.Y., Yan J., Liu T., Chen M.L., Chen Y. (2023). Biochar boosts nitrate removal in constructed wetlands for secondary effluent treatment: Linking nitrate removal to the metabolic pathway of denitrification and biochar properties. Bioresour. Technol..

[4] Zheng H., Wang Z.Y., Deng X., Herbert S., Xing B.S. (2013). Impacts of adding biochar on nitrogen retention and bioavailability in agricultural soil. Geoderma.

[5] Alsewaileh A.S., Usman A.R., Al-Wabel M.I. (2019). Effects of pyrolysis temperature on nitrate-nitrogen (NO_3_^−^-N) and bromate (BrO_3_^−^) adsorption onto date palm biochar. J. Environ. Manag..

[6] Moradzadeh M., Moazed H., Sayyad G., Khaledian M. (2014). Transport of nitrate and ammonium ions in a sandy loam soil treated with potassium zeolite—Evaluating equilibrium and non-equilibrium equations. Acta Ecol. Sin..

[7] Yu G.M., Wang J., Liu L., Li Y., Zhang Y., Wang S.S. (2020). The analysis of groundwater nitrate pollution and health risk assessment in rural areas of Yantai, China. BMC Public Health.

[8] Fseha Y.H., Sizirici B., Yildiz I. (2022). Manganese and nitrate removal from groundwater using date palm biochar: Application for drinking water. Environ. Adv..

[9] Bhatnagar A., Kumar E., Sillanpää M. (2010). Nitrate removal from water by nano-alumina: Characterization and sorption studies. Chem. Eng. J..

[10] Sathishkumar K., Li Y., Sanganyado E. (2020). Electrochemical behavior of biochar and its effects on microbial nitrate reduction: Role of extracellular polymeric substances in extracellular electron transfer. Chem. Eng. J..

[11] Jia W., Yang Y.C., Yang L.Y., Gao Y. (2021). High-efficient nitrogen removal and its microbiological mechanism of a novel carbon self-sufficient constructed wetland. Sci. Total Environ..

[12] Bohdziewicz J., Bodzek M., Wąsik E. (1999). The application of reverse osmosis and nanofiltration to the removal of nitrates from groundwater. Desalination.

[13] Santos G., Lins P., Oliveira L., Silva E., Anastopoulos I., Erto A., Giannakoudakis D., Almeida A., Duarte J., Meili L. (2021). Layered double hydroxides/biochar composites as adsorbents for water remediation applications: Recent trends and perspectives. J. Cleaner Prod..

[14] Vijayaraghavan K., Balasubramanian R. (2021). Application of pinewood waste-derived biochar for the removal of nitrate and phosphate from single and binary solutions. Chemosphere.

[15] Dai Y.J., Wang W.S., Lu L., Yan L.L., Yu D.Y. (2020). Utilization of biochar for the removal of nitrogen and phosphorus. J. Cleaner Prod..

[16] Adesemuyi M.F., Adebayo M.A., Akinola A.O., Olasehinde E.F., Adewole K.A., Lajide L. (2020). Preparation and characterisation of biochars from elephant grass and their utilisation for aqueous nitrate removal: Effect of pyrolysis temperature. J. Environ. Chem. Eng..

[17] Wang Z.H., Guo H.Y., Shen F., Yang G., Zhang Y.Z., Zeng Y.M., Wang L.L., Xiao H., Deng S.H. (2014). Biochar produced from oak sawdust by Lanthanum (La)-involved pyrolysis for adsorption of ammonium (NH_4_^+^), nitrate (NO_3_^−^), and phosphate (PO_4_^3−^). Chemosphere.

[18] Lingamdinne L.P., Choi J.-S., Angaru G.K.R., Karri R.R., Yang J.-K., Chang Y.-Y., Koduru J.R. (2022). Magnetic-watermelon rinds biochar for uranium-contaminated water treatment using an electromagnetic semi-batch column with removal mechanistic investigations. Chemosphere.

[19] Shi Y., Ge Y., Chang J., Shao H.B., Tang Y.L. (2013). Garden waste biomass for renewable and sustainable energy production in China: Potential, challenges and development. Renew. Sustain. Energy Rev..

[20] Zhang Q.C., Wang C.C., Cheng J.H., Zhang C.L., Yao J.J. (2021). Removal of Cr (VI) by biochar derived from six kinds of garden wastes: Isotherms and kinetics. Materials.

[21] Bridgwater A.V. (2012). Review of fast pyrolysis of biomass and product upgrading. Biomass Bioenergy.

[22] Santibañez-Aguilar J.E., Ponce-Ortega J.M., Betzabe González-Campos J., Serna-González M., El-Halwagi M.M. (2013). Optimal planning for the sustainable utilization of municipal solid waste. Waste Manag..

[23] Mukherjee A., Zimmerman A.R., Harris W. (2011). Surface chemistry variations among a series of laboratory-produced biochars. Geoderma..

[24] Abbas Q., Liu G.J., Yousaf B., Ali M.U., Ullah H., Munir M.A.M., Liu R.J. (2018). Contrasting effects of operating conditions and biomass particle size on bulk characteristics and surface chemistry of rice husk derived-biochars. J. Anal. Appl. Pyrolysis.

[25] Liu R.J., Liu G.J., Yousaf B., Abbas Q. (2018). Operating conditions-induced changes in product yield and characteristics during thermal-conversion of peanut shell to biochar in relation to economic analysis. J. Cleaner Prod..

[26] Melo L., Coscione A., Abreu C., Puga A., De C. (2013). Influence of pyrolysis temperature on cadmium and zinc sorption capacity of sugar cane straw-derived biochar. Bioresources.

[27] Ahmadvand M., Soltani J., Hashemi Garmdareh S.E., Varavipour M. (2018). The relationship between the characteristics of Biochar produced at different temperatures and its impact on the uptake of NO_3_^−^–N. Environ. Health Eng. Manag. J..

[28] Jia Y.H., Shi S.L., Liu J., Su S.M., Liang Q., Zeng X.B., Li T.S. (2018). Study of the effect of pyrolysis temperature on the Cd_2_^+^ adsorption characteristics of biochar. Appl. Sci..

[29] Divband Hafshejani L., Hooshmand A., Naseri A.A., Mohammadi A.S., Abbasi F., Bhatnagar A. (2016). Removal of nitrate from aqueous solution by modified sugarcane bagasse biochar. Ecol. Eng..

[30] Viglašová E., Galamboš M., Danková Z., Krivosudský L., Lengauer C.L., Hood-Nowotny R., Soja G., Rompel A., Matík M., Briančin J. (2018). Production, characterization and adsorption studies of bamboo-based biochar/montmorillonite composite for nitrate removal. Waste Manag..

[31] Zhou L., Xu D.F., Li Y.X., Pan Q.C., Wang J.J., Xue L.H., Howard A. (2019). Phosphorus and nitrogen adsorption capacities of biochars derived from feedstocks at different pyrolysis temperatures. Water..

[32] Hollister C.C., Bisogni J.J., Lehmann J. (2013). Ammonium, Nitrate, and Phosphate Sorption to and Solute Leaching from Biochars Prepared from Corn Stover (*Zea mays* L.) and Oak Wood (*Quercus* spp.). J. Environ. Qual..

[33] He X.Y., Liu Z.X., Niu W.J., Yang L., Zhou T., Qin D., Niu Z.Y., Yuan Q.X. (2018). Effects of pyrolysis temperature on the physicochemical properties of gas and biochar obtained from pyrolysis of crop residues. Energy.

[34] Al-Wabel M.I., Al-Omran A., El-Naggar A.H., Nadeem M., Usman A.R.A. (2013). Pyrolysis temperature induced changes in characteristics and chemical composition of biochar produced from conocarpus wastes. Bioresour. Technol..

[35] Kwak J.H., Md-Islam S., Wang S., Messele S.A., Naeth M.A., El-Din M.G., Chang S.X. (2019). Biochar properties and lead(II) adsorption capacity depend on feedstock type, pyrolysis temperature, and steam activation. Chemosphere.

[36] Rahmani A., Mousavi H.Z., Fazli M. (2010). Effect of nanostructure alumina on adsorption of heavy metals. Desalination.

[37] Chintala R., Mollinedo J., Schumacher T.E., Papiernik S.K., Malo D.D., Clay D.E., Kumar S., Gulbrandson D.W. (2013). Nitrate sorption and desorption in biochars from fast pyrolysis. Microporous Mesoporous Mater..

[38] Li R.H., Wang J.J., Zhou B.Y., Awasthi M.K., Ali A., Zhang Z.Q., Gaston L.A., Lahori A.H., Mahar A. (2016). Enhancing phosphate adsorption by Mg/Al layered double hydroxide functionalized biochar with different Mg/Al ratios. Sci. Total Environ..

[39] Mazarji M., Aminzadeh B., Baghdadi M., Bhatnagar A. (2017). Removal of nitrate from aqueous solution using modified granular activated carbon. J. Mol. Liq..

[40] Zhang J., Lu W.J., Zhan S.Y., Qiu J.M., Wang X.M., Wu Z.D., Li H., Qiu Z.M., Peng H.L. (2021). Adsorption and mechanistic study for humic acid removal by magnetic biochar derived from forestry wastes functionalized with Mg/Al-LDH. Sep. Purif. Technol..

[41] Chen X., Dai Y.H., Fan J., Xu X.Y., Cao X.D. (2021). Application of iron-biochar composite in topsoil for simultaneous remediation of chromium-contaminated soil and groundwater: Immobilization mechanism and long-term stability. J. Hazard. Mater..

[42] Ahmadi M., Kouhgardi E., Ramavandi B. (2016). Physico-chemical study of dew melon peel biochar for chromium attenuation from simulated and actual wastewaters. Korean J. Chem. Eng..

[43] Rudi N.N., Muhamad M.S., Chuan L.T., Alipal J., Omar S., Hamidon N., Abdul Hamid N.H., Mohamed Sunar N., Ali R., Harun H. (2020). Evolution of adsorption process for manganese removal in water via agricultural waste adsorbents. Heliyon.

