# Supplementary figures and images for: Nitrate-Nitrogen Adsorption Characteristics and Mechanisms of Various Garden Waste Biochars
Source: Materials (Basel). 2023 Aug 21;16(16):5726. doi: 10.3390/ma16165726 (PMC10456472; doi:10.3390/ma16165726)

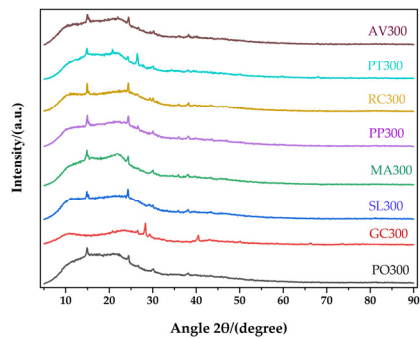

(a)

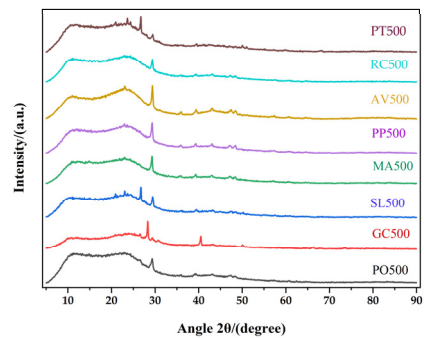

(b)

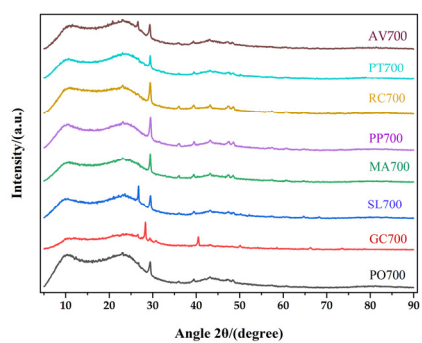

(c)

Figure S1. XRD patterns of different biochars.

Supplement: Supplementary file 1 [file materials-16-05726-s001.zip › Figure S1.pdf]

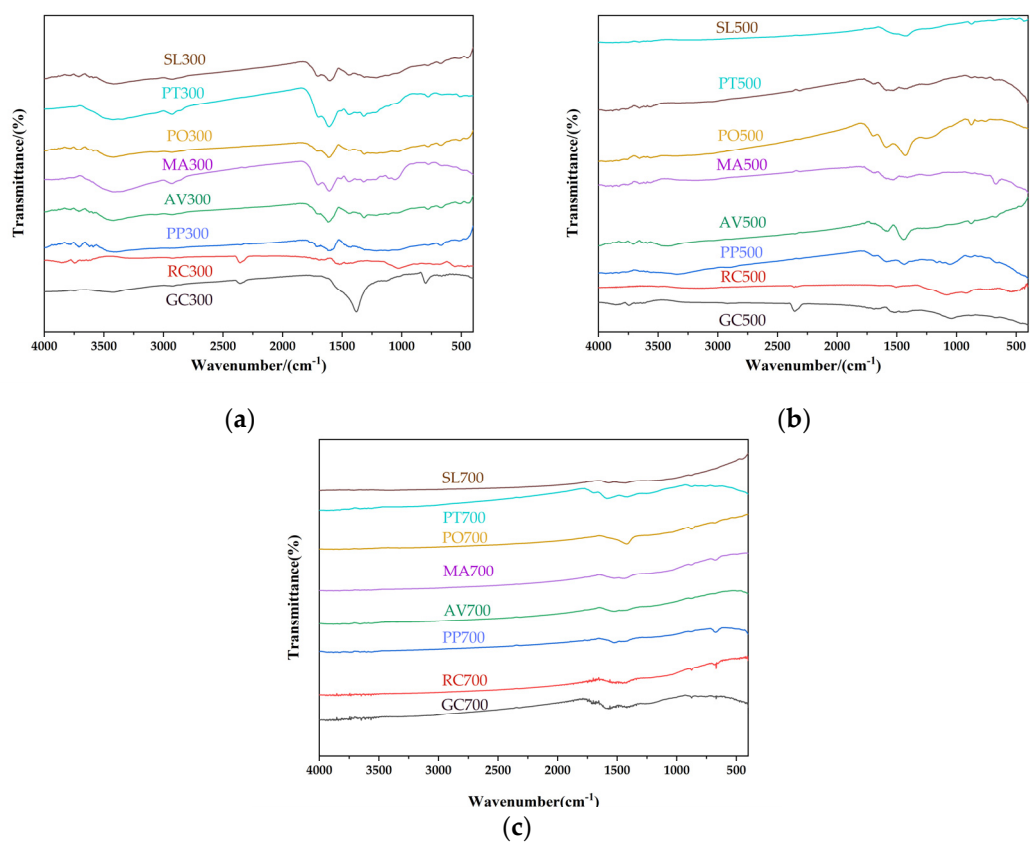

**Figure S2.** FTIR spectra of different biochars.

Supplement: Supplementary file 1 [file materials-16-05726-s001.zip › Figure S2.pdf]

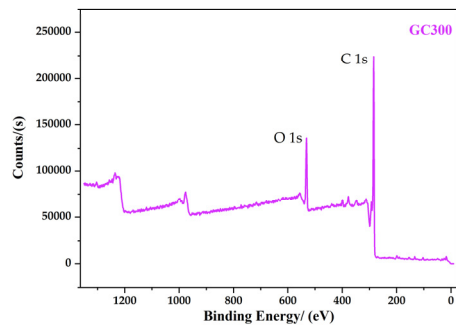

(a)

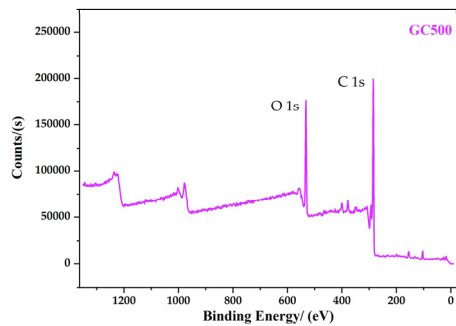

(b)

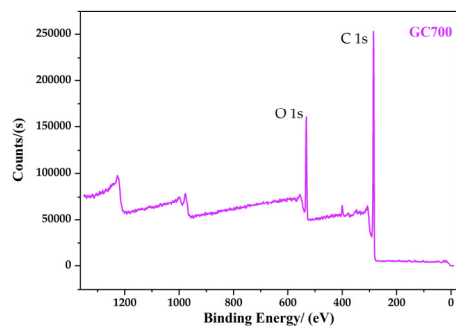

(c)

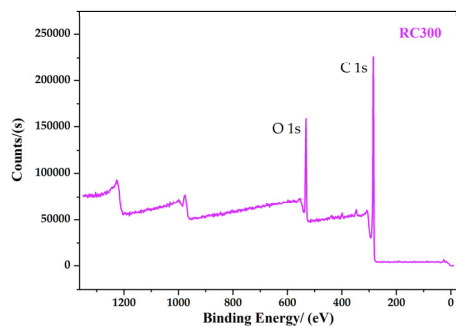

(d)

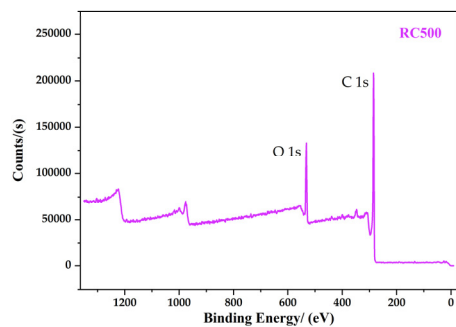

(e)

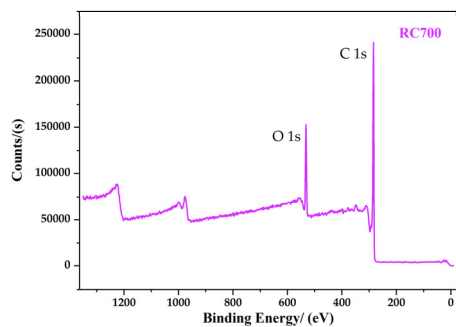

(f)

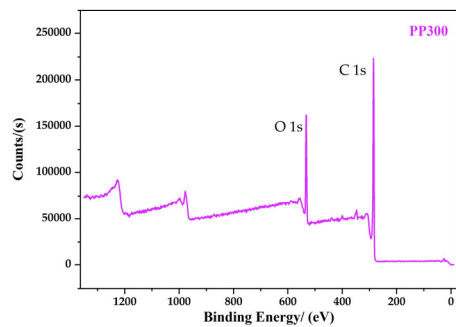

(g)

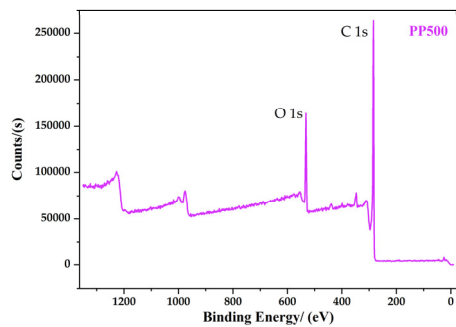

(h)

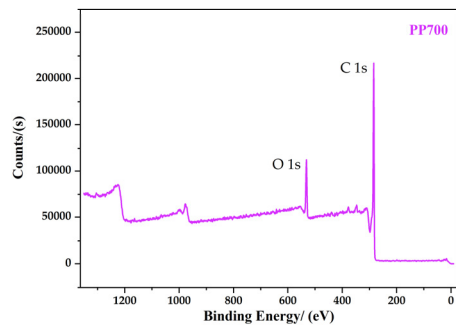

(i)

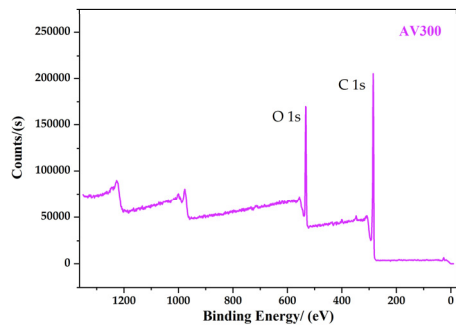

(j)

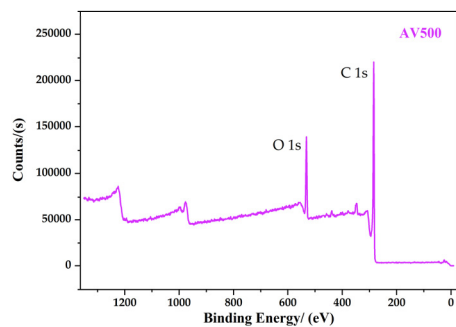

(k)

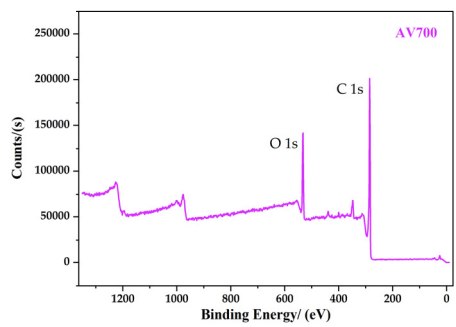

(l)

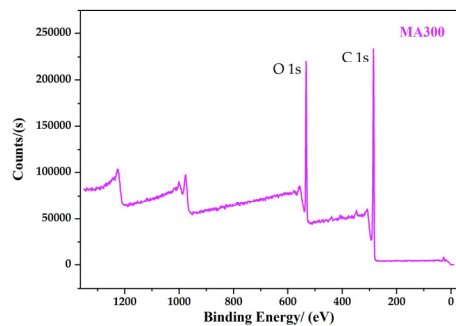

(m)

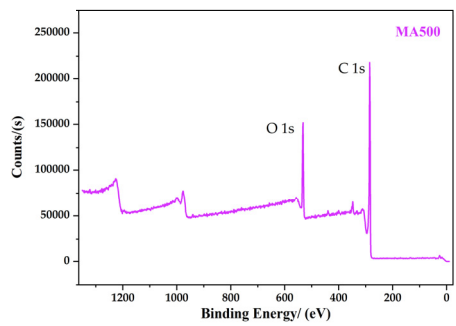

(n)

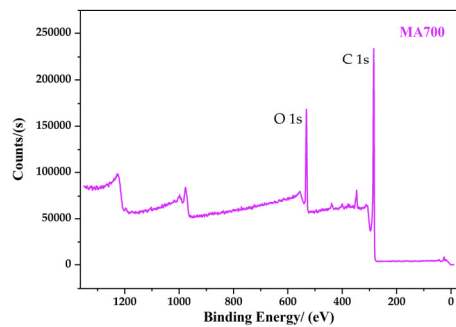

(o)

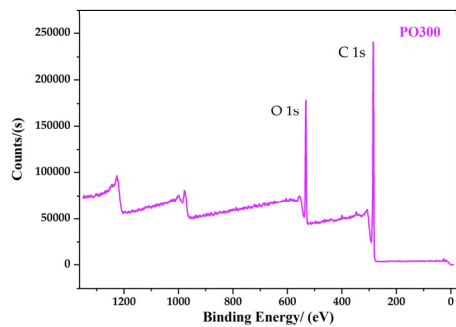

(p)

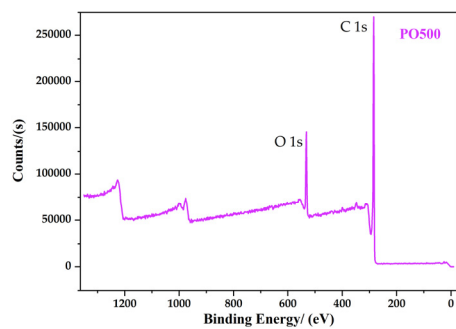

(q)

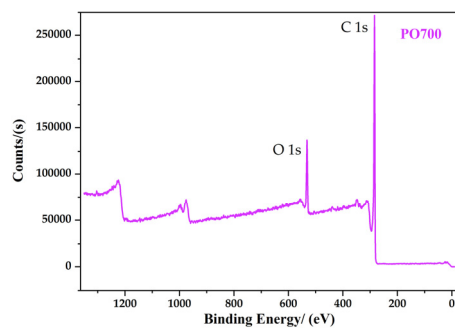

(r)

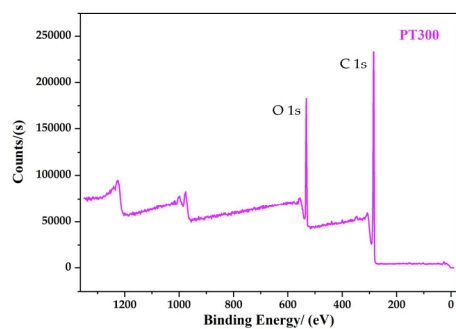

(s)

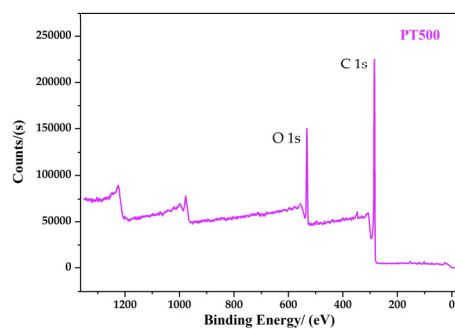

(t)

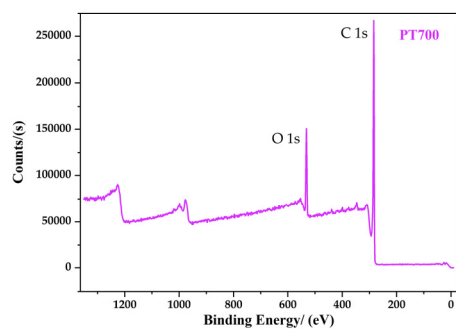

(u)

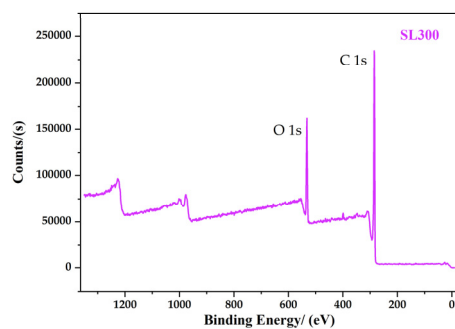

(v)

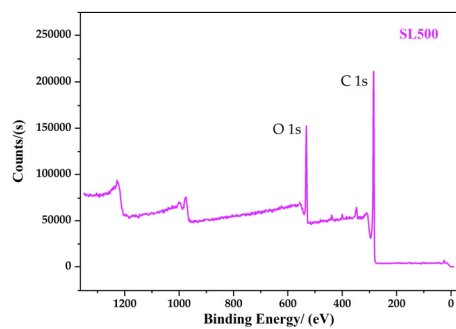

(w)

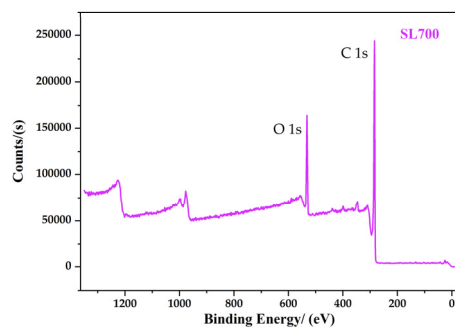

(x)

Figure S3. The wide scale XPS spectra of different biochars.

Supplement: Supplementary file 1 [file materials-16-05726-s001.zip › Figure S3.pdf]

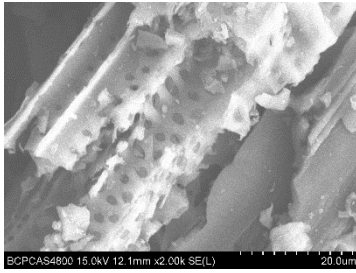

GC300

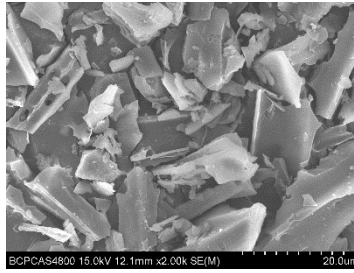

GC500

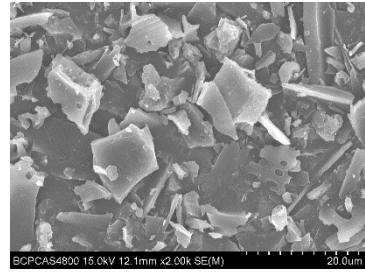

GC700

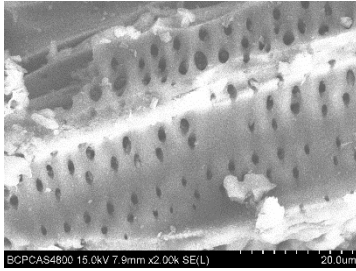

RC300

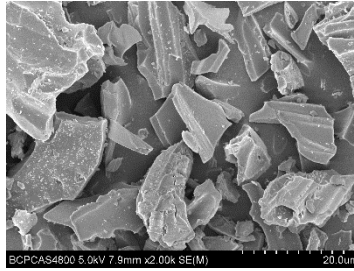

RC500

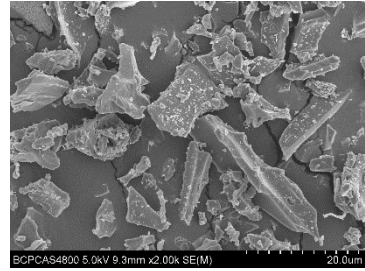

RC700

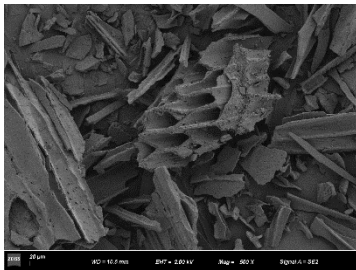

PP300

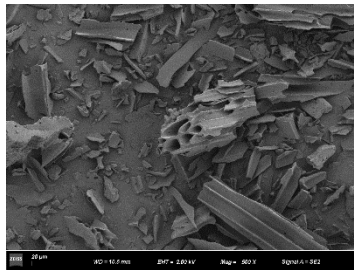

PP500

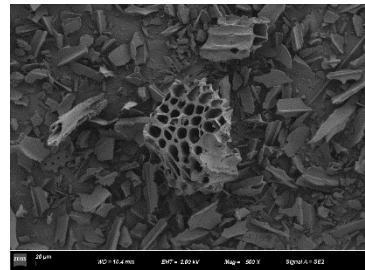

PP700

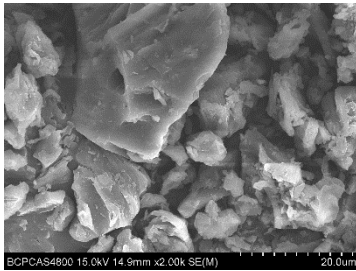

AV300

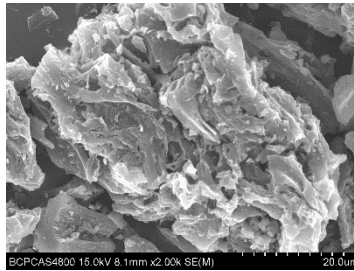

AV500

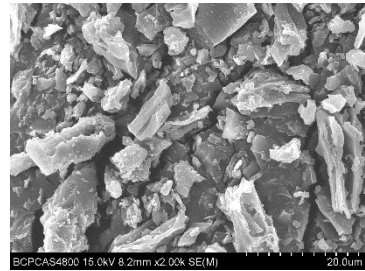

AV700

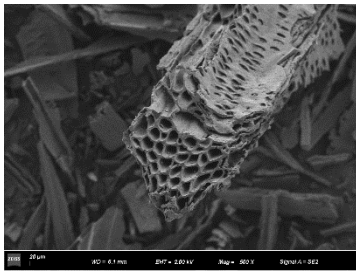

MA300

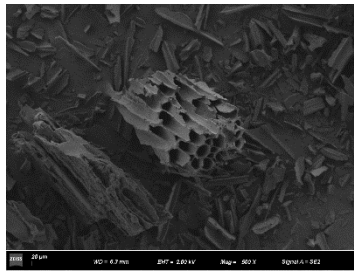

MA500

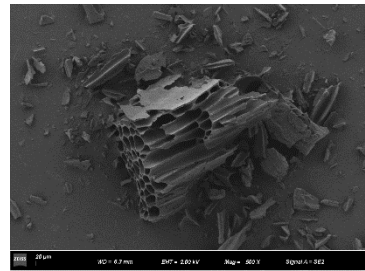

MA700

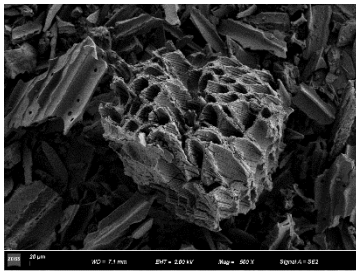

PO300

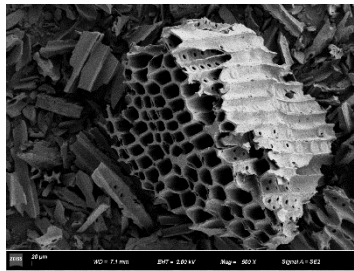

PO500

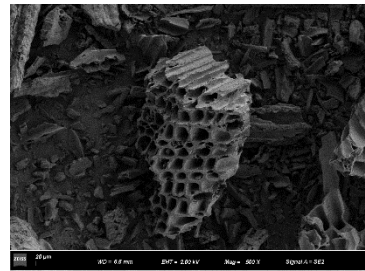

PO700

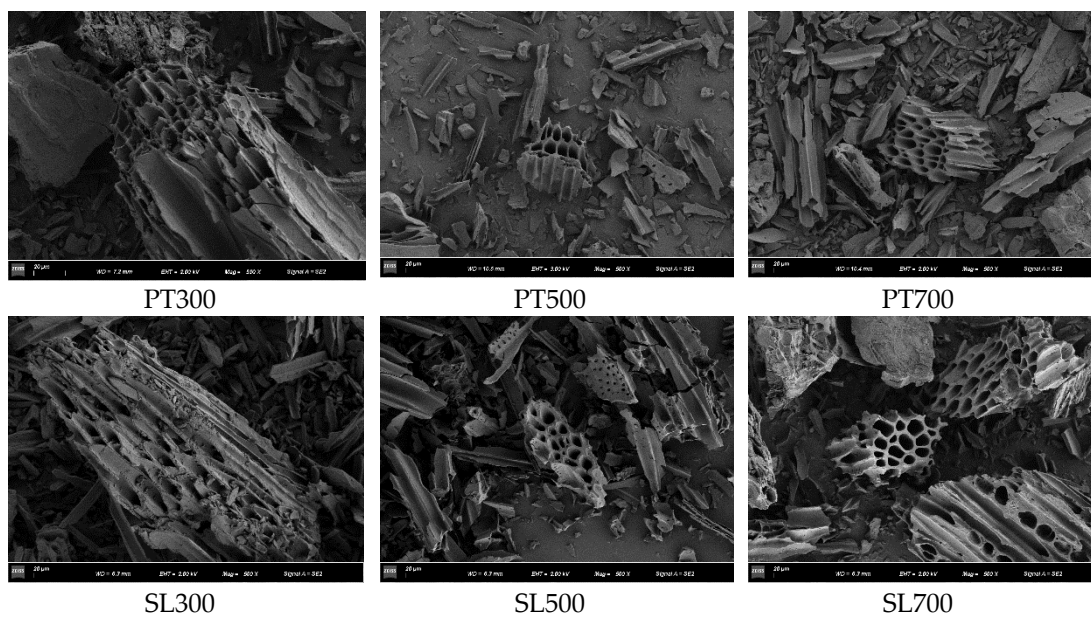

**Figure S4.** SEM of different biochars.

Supplement: Supplementary file 1 [file materials-16-05726-s001.zip › Figure S4.pdf]

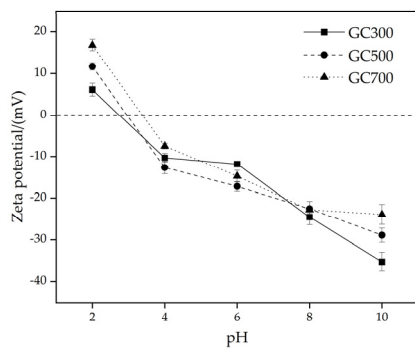

(a)

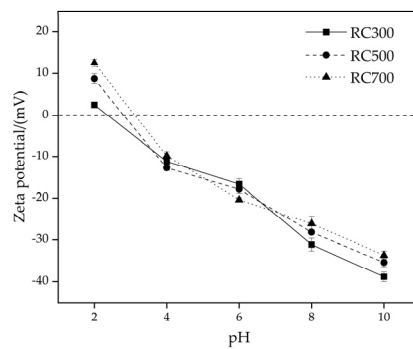

(b)

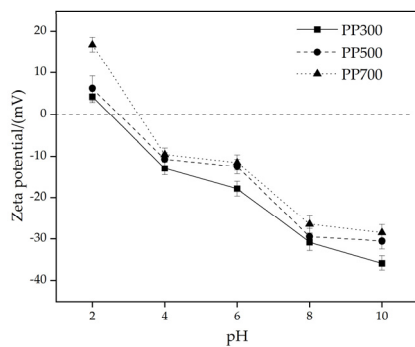

(c)

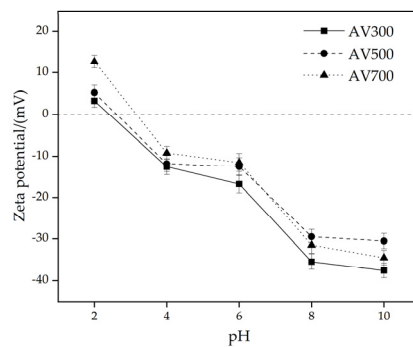

(d)

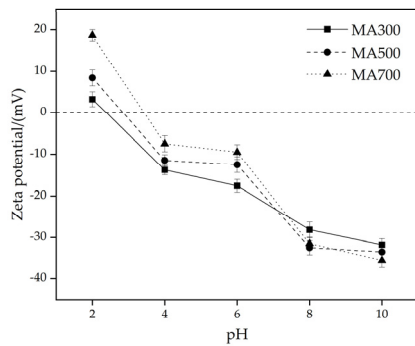

(e)

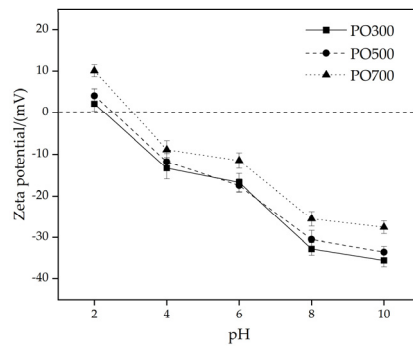

(f)

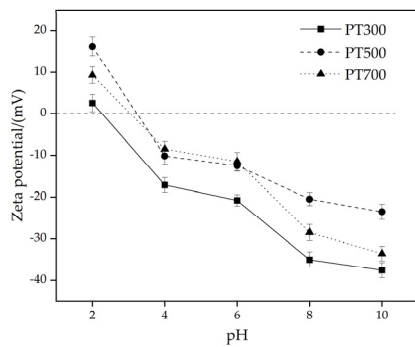

(g)

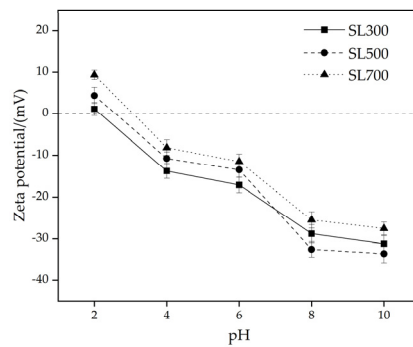

(h)

Figure S5. Zeta potential of different biochars.

Supplement: Supplementary file 1 [file materials-16-05726-s001.zip › Figure S5.pdf]
